# Justinian Rhinotmetos: A Byzantine Rhinoplasty?

**DOI:** 10.7759/cureus.68089

**Published:** 2024-08-29

**Authors:** Matthew D Turner, Michael J Lawson

**Affiliations:** 1 Emergency Medicine, Penn State Health Milton S. Hershey Medical Center, Hershey, USA; 2 Dermatology, Brooke Army Medical Center, San Antonio, USA

**Keywords:** plastic and reconstructive surgery, byzantine history, history of surgery, indian rhinoplasty, reconstructive rhinoplasty

## Abstract

In the eighth century, Justinian II was overthrown from his position as ruler of the Byzantine Empire. The young ruler’s nose was amputated, and he was exiled. Ten years later, he regained the throne in a bloody coup. For decades, researchers have debated if Justinian also regained his nose through the ancient Indian rhinoplasty surgical technique, largely based on the Carmagnola statue in modern Venice. While a fascinating possibility, we ultimately conclude that it is highly unlikely that this ever occurred.

## Introduction and background

The two reigns of Justinian II

Justinian II was born in 669 AD as the heir to Constantine IV. At the young age of 16, he first rose to the Byzantine throne when his father died of dysentery at 33 [1]. The newly crowned *Basileus *(“Emperor”) of the Byzantine Empire [1] inherited a realm in turmoil. The past century had been a tumultuous period for Byzantium, with recurrent bouts of plague, natural disasters, encroaching enemies, the loss of some of its wealthiest provinces, and depopulation [2]. Despite this, the early days of Justinian’s reign gave the empire a brief respite; within a few years, he swiftly dispatched his commanding general Leonitus to defeat his enemies in present-day Georgia and Greek Macedonia [1]. Justinian quickly focused on strengthening his position, both militarily, by building up the empire’s defenses, and symbolically. The *solidus *coin was the lynchpin in the Byzantine economy, and so it was in the empire’s interest to maintain the coinage’s legitimacy at all costs. Any changes to the *solidus *in the past had been very rare, and so it was an extremely radical innovation when Justinian unveiled his changes to the currency [3]. For the very first time, Justinian featured an image of Christ on his coinage, while placing an image of himself on the reverse of the coin, adopting the title *Servus Christi*, “servant of Christ.” In this way, the young Basileus portrayed himself as an object of divine will [2], displaying a canny knowledge of the importance of symbology from a young age.

Despite Justinian’s promising beginning, the Byzantine Empire, hollowed out and lacking resources from decades of conflict and instability, began to crumble once more [2]. In 692 AD, Justinian broke a 14-year-old treaty with the Arabs; his general Leonitus was badly defeated by Arab forces at the Battle of Sebastopolis and imprisoned by the *Basileus *as punishment [1]. In addition to the military disaster, heavy taxation and a consistent disregard for the Byzantine senate significantly cut into Justinian’s support at home [4]. Justinian’s reign finally collapsed after little more than a decade. Leonitus escaped from prison and led a major rebellion against the *Basileus *[1], supported by the Blues, a powerful aristocratic party [4]. Justinian was swiftly captured, and at the age of 26 [4], dragged to the Hippodrome, where thousands of his former subjects cheered as the Basileus was subjected to *rhinokopia* (cutting off the nose) and *glossotomia* (slitting of the tongue) [1].

Mutilation, specifically rhinectomy, was a common punishment in the Byzantine court and one that Justinian would have likely already been familiar with. Justinian’s uncles had had their noses slit by his own father, to preserve his own political power [4]. A few decades earlier, Heraklonas had suffered a similar punishment, where the politician had had his nose cut off and was subsequently exiled to Rhodes [4]. It was the primary penalty used against both rebels and members of the royal family that had been removed by the throne-as *Basileus*, Justinian was expected to conform to an ideal of “perfection”-with such a visible mutilation, political power was barred to him forever. Strangely, this was regarded as a lenient punishment in the world of Byzantine politics, as the alternative would have been execution [5]. The nose was also seen to be somewhat reflective of one’s inner character, tradition held that both Christ and Paul the Apostle had had hooked noses, and the Roman Emperor Augustus was said to have had a "Roman nose" that was indicative of a “great-souled man” [6]. Justinian’s mutilation forever barred him from being associated with such beloved figures [6]. On a wider scale, by the time of the seventh century, legalized rhinectomy appears to have been widespread outside the royal court; many Byzantine chroniclers record it being used to punish gambling and other illegal activities [5].

After his brutal punishment, Justinian was exiled to Cherson in the Crimean Peninsula. Banished away from Constantinople and physically deformed, his enemies were sure that he could never regain political power [1]. This was a fatal mistake. The former *Basileus *rapidly recovered from his wounds; he appears to have suffered no long-term issues from the mutilation done to his tongue [4] and quickly set about re-establishing a base of political power. He escaped Cherson and fled to the north, where he found refuge and allies among the Khazars of northern Crimea [4]. He married the sister to the Khagan, the leader of the Khazars, and rechristened her as Theodora, a deliberate reference to the wife of the legendary Justinian I [1]. His strength and popularity quickly grew in the region, so much so that the governor of Cherson attempted to have him assassinated [1]. Justinian strangled the two would-be assassins with his bare hands and fled to modern-day Bulgaria with a small force of loyal Khazars [1]. There, he allied with the local Bulgars and marched on Constinatinople at the head of a furious army [1]. In the spring of 705 AD, he laid siege to the empire’s capital, and after three days of stalemate, stealthily entered and captured the crucial city [7].

Ironically, Leonitus, his former general and usurper, had himself been overthrown seven years previously. He had also been subject to *rhinokopia *and then banished to a monastery [1]. The current *Basileus*, Tiberius Apsimar, a former admiral [1], quickly fled the city, although he was captured a few weeks later [7]. Justinian, now popularly called Justinian *Rhinometos*, “Justinian with the cut-off nose” [1], set about his second reign with a bloody vengeance. Leonitus was taken from his monastery and, along with Apsimar, dragged into the Hippodrome where Justinian proclaimed himself Emperor to a throng of thousands. Placing a foot on the neck of each man, Justinian declared victory over the “asp and lion” [1]. The two men were then swiftly beheaded, and thousands of their followers purged via hanging, beheading, and drowning. Patriarch Kallinikos, who had crowned both of the usurpers, was blinded and then banished to Rome [1].

Justinian’s second reign lasted only six years but was so bloody and destructive that it left the empire on the very edge of collapse [2]. Eroded by invasion, purges [1], revolts, a collapsing bureaucracy, and loss of organization [2], the end finally came in 711 AD, when rebel forces captured Justinian as he was traveling outside Constantinople. He was beheaded, his lifeless body hurled into the sea, and his head proudly displayed in tours across the empire, where his death was widely celebrated [1].

## Review

The Carmagnola

While multiple written sources agree that Justinian II suffered rhinectomy at the end of his first reign, and he was widely known as Justinian *Rhinotmetos *throughout his second; there are no confirmed contemporary images of the *Basileus*’ mutilation. The coinage throughout Justinian’s second reign shows no image of rhinectomy or nasal trauma whatsoever; he is displayed with a “well-shaped normal nose” and narrow, angular features [1]. No other portraits from his reign appear to display the reported mutilation [6]. A single chronicler named Agnellus of Ravenna reported that the Basileus returned to power with an artificial golden nose [1]. However, Agnellus has a reputation of being an unreliable historical source, writing approximately 200 years after Justinian’s reign [4]. In addition to this, he claimed that Justinian had prosthetic “golden ears” as well [1], asserting that the *Basileus’s *ears had also been amputated, which is not corroborated by any other source [4].

However, a contemporary depiction of Justinian II’s nasal deformity may have survived to the present day. In a 1979 paper, researchers theorized that the Carmagnola head of Venice may represent Justinian II [1], a theory first proposed by the German archaeologist Richard Delbrück in 1913 [8]. However, the 1979 researchers proposed that the Carmagnola statue also displayed evidence that a rhinoplasty procedure had been used to repair Justinian’s rhinectomy, an unprecedented claim [1].

Before this can be fully discussed, the Carmagnola statue must be addressed. In its current form, the Carmagnola statue is the statue of a “finely sculpted, red, porphyry head undoubtedly represent[ing] a Byzantine emperor” that is mounted on the balcony of the San Marco cathedral in Venice [1]. The Carmagnola originated in Constantinople, where it is theorized that it was one of the Tetrarchs, four statues that stood in the city’s *Philadelphion *[9]. After the city fell to forces of the Fourth Crusade in 1204, Venetian forces triumphantly brought back the head of one of the statues and mounted it on the balcony of San Marco Cathedral overlooking the *Piazzetta*. The placement was intentional; the *Piazzetta *was the traditional place of executions in medieval Venice, and even as late as 1611, visitors to the city complained of the stench from the severed heads of prisoners mounted there [9]. By mounting the severed head of a statue overlooking the executions, taken as booty from an enemy city, the Venetians made a bold statement of political power, following in the ancient Roman tradition of proudly displaying the severed heads of fallen foes [9]. The Carmagnola statue eventually earned its name from Francesco Bussone de Carmagnola, an accused criminal who was tortured and beheaded beneath the stone head’s gaze in the *Piazzetta *in 1432 [8].

**Figure 1:**
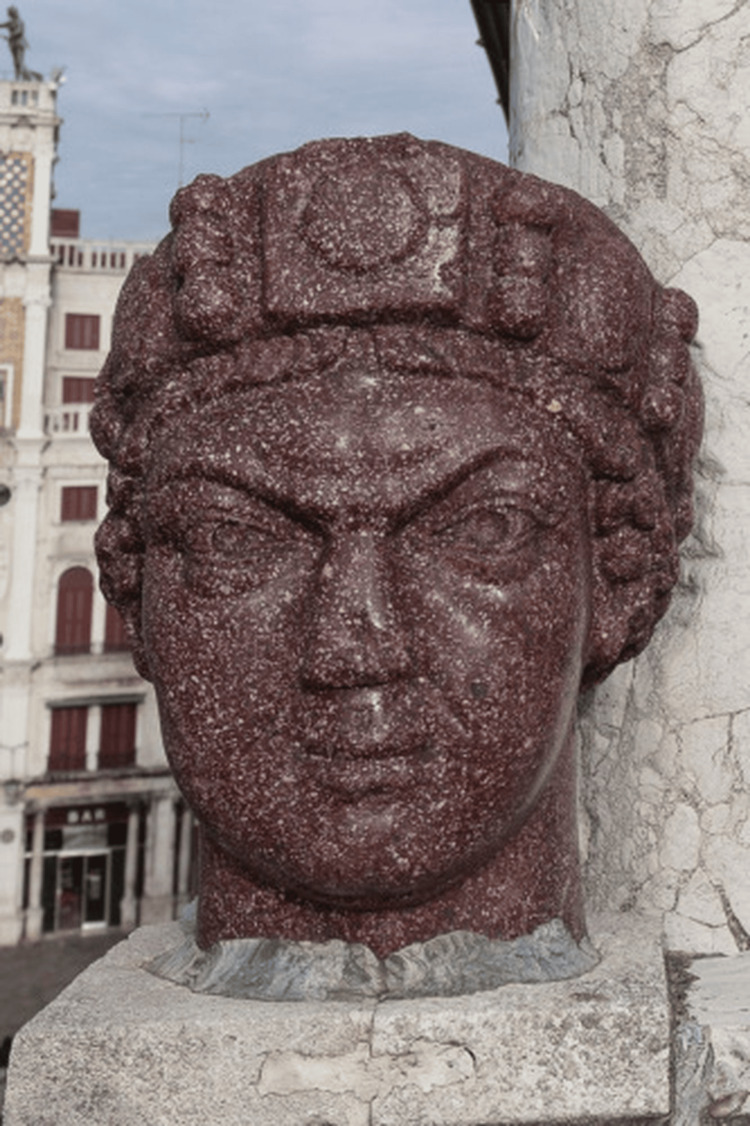
The Carmagnola head. Note the damage to the nasal bridge This picture was used under Creative Commons Attribution 4.0 International (CC BY 4.0). Source: [10]

**Figure 2:**
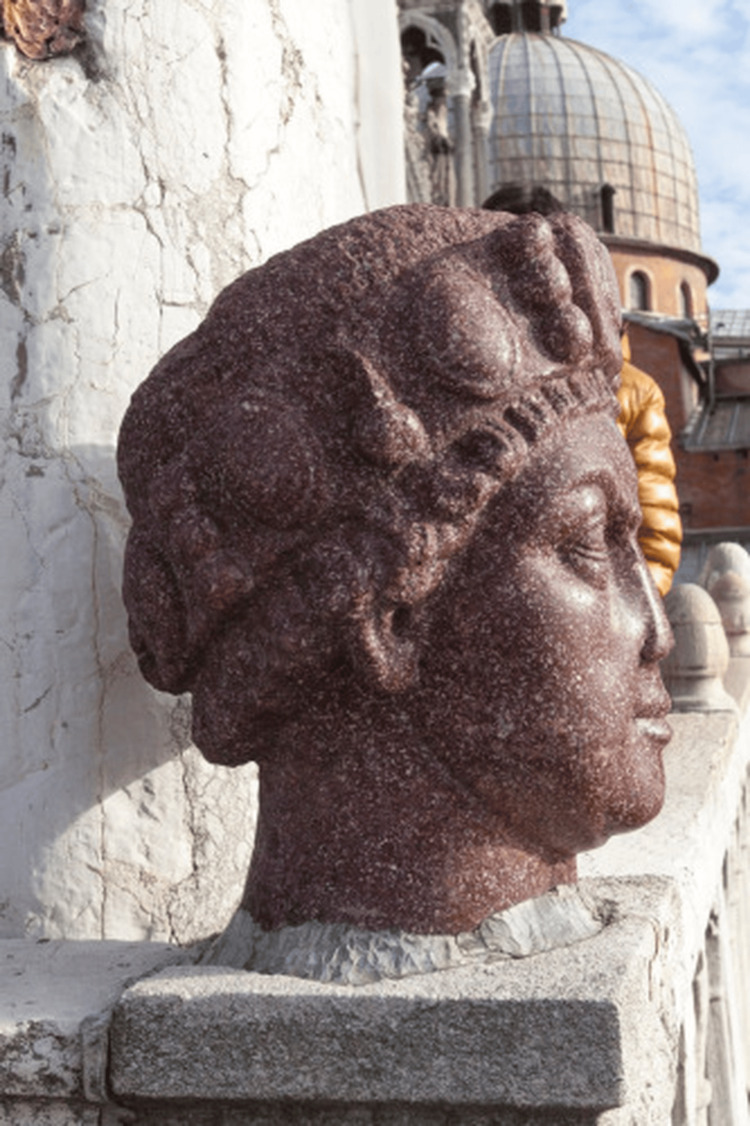
The Carmagnola head seen in profile. Note the flattened appearance of the nose Used under Creative Commons Attribution 4.0 International (CC BY 4.0). Source: [10]

The Carmagnola is particularly notable for its use of red porphyry, a “hard and beautiful material which was reserved for imperial purposes” [1], leading multiple sources to conclude that it depicts a Byzantine emperor [8]. The statue’s nose is unusual; there is a gouge in the front section of the nose, which has an “extremely flat profile, without signs of any further damage… nor does it have the mashed and flattened appearance of a broken nose” [1]. The crudeness of the nose, which has no notable alar curve, no nostril openings, and lacks “any of the normal undulations and subtleties of the normal nose,” is highly inconsistent with the delicately chiseled features of the rest of the statue [1]. Delbrück concluded that this damage to the statue’s nose was not accidental or inflicted later but an intentional choice on the part of the sculptor; he ultimately decided that this statue represented Justinian II [8]. In the 1979 paper, researchers again investigated the statue and agreed that the abnormalities to the Carmagnola’s nose appeared to be intentional; in addition to this, there are “irregularities” to the statue’s forehead as well, suggesting the presence of scars [1]. Figures 1 and 2 depict the Carmagnola in its current form.

Delbrück suggested that the abnormalities found on the Carmagnola, if it indeed represents Justinian II, could represent a previously undocumented rhinoplasty procedure that the *Basilieus *had received, a possibility that the 1979 paper also acknowledges [1].

"Indian rhinoplasty"

Contemporary Byzantine surgery, which provides a direct link between medicine of the ancient world and modern Western medicine, was surprisingly advanced [11]. The empire had a professional class of public and private physicians [12] that were frequently allowed to perform anatomic studies [13], making them well-versed in surgical procedures. Paul of Aegina, writing nearly a century before Justinian II, dedicated a great deal of his works to surgical procedures, including a number of plastic surgery operations such as gynecomastia in males and corrective rhacosis for excessive scrotal skin [11]. Byzantine surgeons could also perform tracheotomies, perform lithotripsies of the bladder, separate conjoined twins, treat inguinal hernias, and even perform reconstructive surgeries of the face [13]. Paul of Aegina wrote extensively on splinting of nasal and mandibular fractures, as well as corrective surgeries of the eyelids [14]. Oribasius Pergamenus, writing over 300 years before Justinian II, even described surgical reconstruction of the face [14].

However, while Byzantine rhinology was advanced capable of treating rhinitis, epistaxis, polyps, cancer, bruises, and nasal fractures [14], there is no evidence that the Byzantines ever had any means of imitating rhinoplasty [1].

While the medicine of the Byzantine Empire lacked any rhinoplasty at the time of Justinian II, rhinoplasty was already an ancient technique in India. First described in the *Samhita*, written around 1000-800 BCE, the legendary Indian physician Sushruta described the technique, where a physician used an adjacent flap of skin from the cheek to repair a destroyed nose [15]. Although rhinectomy was a common punishment throughout the ancient world, often used as a legal punishment for adultery or against political opponents, and documented in sources such as as the Hammurabi code, it was especially widespread in ancient India [16]. The epic poem Ramayana describes Lakshan cutting off the nose of the evil Lady Supanakha, which appeared to provide divine sanction for the custom [17]. It was also commonly performed in warfare; in 1770, when the city of Kirtipoor fell to King Priviti Narayan, the furious king amputated the noses of all 865 surviving male inhabitants and mockingly changed the city’s name to Naskatapoor, “the city without noses” [18].

**Figure 3:**
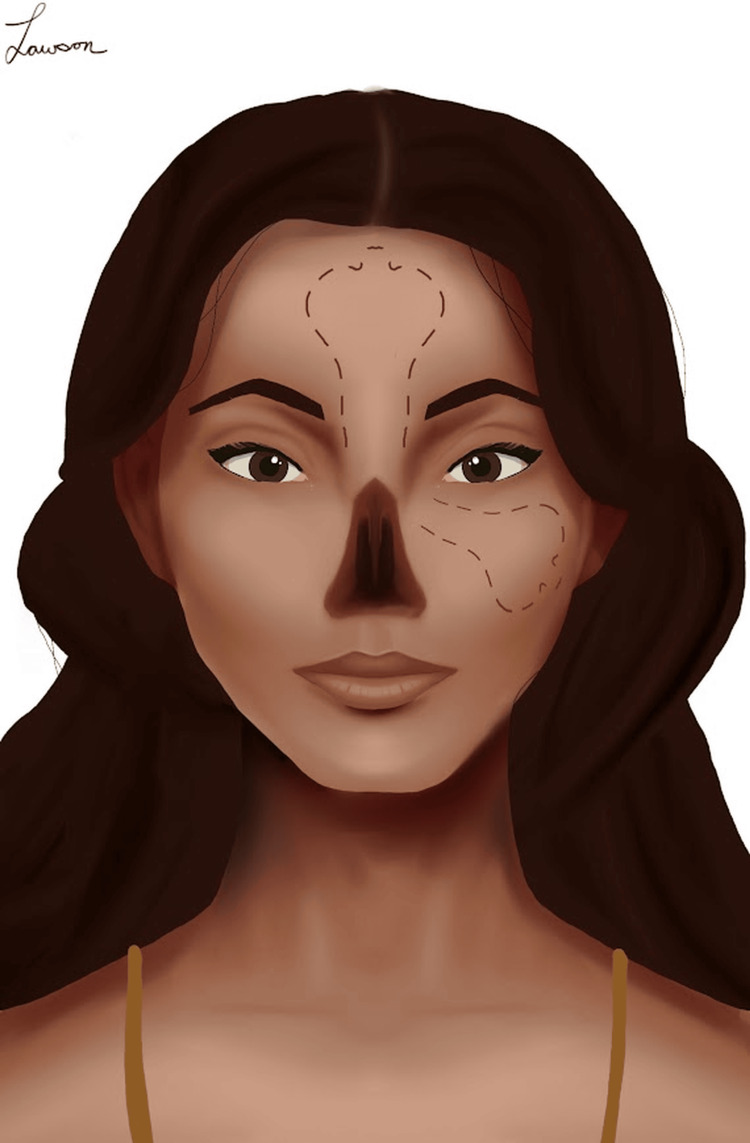
Example of the various types of Indian rhinoplasty. The incision on the cheek shows the traditional technique described by Sushruta, while the forehead incision shows the more modern technique often referred to as "Indian rhinoplasty" Image drawn by Dr. Michael Lawson, one of the authors of this paper

In an environment where rhinectomy was so common, the ancient Indians quickly adopted Sushruta’s rhinoplasty technique. A special caste of priests known as the Koomas took over the process in most regions, although some well-known families also performed the surgery, passing down the art of rhinoplasty from father to son in strict secrecy [18]. Over the centuries, Indian rhinoplasty transitioned from using skin flaps from the cheek to skin flaps taken from the forehead [17], as shown in Figure 3. While a number of medieval Italian surgeons performed rhinoplasty, interest gradually faded away [15]. It wasn’t until the early 19th century that knowledge of the “Indian technique” came back to the forefront of European medicine, laying the foundation for a new age of plastic surgery in the West [17].

Justinian II and rhinoplasty

Delbrück argued that the abnormalities on the Carmagnola’s nose indicate that Justinian II may have had his prior rhinectomy repaired through the ancient Indian technique of rhinoplasty, an argument supported by the evidence of scars on the sculpture’s forehead [1]. While there is a remote possibility of this, it appears highly unlikely. Although the Byzantine Empire was a crossroads of medicine, from the ancient world of Greece and Rome to modern western medicine [12], there is no evidence that rhinoplasty was ever practiced in this civilization [1]. If anything, contemporary Western medicine, still heavily influenced by the writings of Galen, held that there was absolutely no possibility that an amputated nose could be regrown or repaired, an opinion practically universally held for centuries [8]. If the *Basileius *of the Byzantine Empire had somehow had his rhinectomy repaired, there would likely have been a far greater presence of this in the historical records.

Secondly, it remains doubtful if the damage to the Carmagnola’s nose was intentional. When the capital of the Byzantine Empire fell to the Fourth Crusade in 1204, the crusaders took out their fury on the statues of the Tetrarchs, which were considered symbolic of the city’s resistance. The statues received symbolic blows, sustaining damage to their nose, their ears clipped off, their eyes gouged out, as a symbolic way to “deny them access to the world by immobilizing them and traumatizing their sensory organs” [9]. Although it was not one of the Tetrarchs, a nearby statue was later noted by a Russian pilgrim to have had its nose cut off by the crusaders [9]. It is possible that the Carmagnola’s deformed nose was never intentionally made by its sculptor but inflicted by one of the soldiers of the Fourth Crusade when the statue was beheaded and then transported back to Venice. In addition to this, it is highly unlikely that Justinian II would have wanted the “humiliating marks of his disfigurement portrayed in his imperial portrait in stone” [1]. Throughout his multiple reigns, he carefully managed his image in official portraiture, taking pains to depict himself as a regal presence that was both subservient to and divinely ordained by Christ [3]. Justinian’s official coinage never depicted any hint of his disfigurement [6]. It appears extremely unlikely that a paranoid emperor would allow any depictions of himself that reminded of his prior rhinectomy and exile [1]. Even the scarring on the Carmagnola’s forehead could simply be the remnants of a Greek cross carved into the forehead, a common convention in statues at the time, rather than scar tissue left from a prior Indian rhinoplasty [1].

Finally, there is some debate over whether the Carmagnola statue actually depicts Justinian II. During his second reign, Justinian II is depicted with a beard on all of his coinage, something that the clean-shaven Carmagnola lacks [1]. The statue may actually depict Justinian I “the Great”, who ruled over a century earlier [19]. One interesting theory holds that both emperors may be represented in the statue; the statue may have originally been of Justinian I but then ground down and altered to mimic Justinian II’s features. Justinian II was fascinated with his namesake, even baptizing his wife with the name Theodora after Justinian I’s famous bride [1]. Unfortunately, this theory remains impossible to prove.

## Conclusions

While it remains an interesting possibility, it is highly unlikely that Justinian II of the Byzantine Emperor had any sort of rhinoplasty performed. While such techniques were possible in India at the time, there is no evidence that they traveled to the contemporary Byzantine Empire. In addition to this, none of Justinian’s portraiture depicts any evidence of his mutilation, and there is no mention in the literature of any sort of rhinoplasty. The sole piece of evidence for Justinian II’s alleged rhinoplasty is a single statue that may not actually depict the Basileus at all. While impossible to conclusively prove, it appears highly unlikely that this interesting historical footnote in the annals of plastic surgery ever occurred.

## References

[1] Remensnyder JP, Bigelow ME, Goldwyn RM. (1979). Justinian II and Carmagnola: a Byzantine rhinoplasty?. J Plast Recontr Surg.

[2] Humphreys M (2011). Images of authority? Imperial patronage of icons from Justinian II to Leo III. An Age of Saints? Power, Conflict and Dissent in Early Medieval Christianity.

[3] Humphreys M (2013). The 'war of images' revisited: Justinian II's coinage reform and the caliphate. Numis Chron.

[4] Laes C (2019). Power, infirmity and ‘disability’. Five case stories on Byzantine emperors and their impairments. Byzantinoslavica-Revue internationale des Etudes Byzantines.

[5] Lascaratos J, Dalla-Vorgia P (1997). The penalty of mutilation for crimes in the Byzantine era (324-1453 A.D.). Int J Risk Saf Med.

[6] Baldwin B (1981). Physical descriptions of Byzantine emperors. Byzantion.

[7] Head C (1969). On the date of Justinian II's restoration. Byzantion.

[8] Bondio MG (2017). On the function, utility, and fragility of the nose: early modern patients and their surgeons. Nuncius.

[9] Barry F (2010). Disiecta membra: Ranieri Zeno, the imitation of Constantinople, the spolia style, and justice at San Marco. San Marco, Byzantium, and the myths of Venice.

[10] (2024). Carmagnola (head). https://catalogo.beniculturali.it/detail/Veneto/ArchaeologicalProperty/CRV-RA_0013525.

[11] Papadakis M, Manios A, de Bree E, Trompoukis C, Tsiftsis DD (2010). Gynaecomastia and scrotal rhacosis: two aesthetic surgical operations for men in Byzantine times. J Plast Reconstr Aesthet Surg.

[12] Ramoutsaki IA, Papadakis CE, Ramoutsakis IA, Helidonis ES (2002). Therapeutic methods used for otolaryngological problems during the Byzantine period. Ann Otol Rhinol Laryngol.

[13] Lascaratos JG, Tsiamis C, Kostakis A (2003). Surgery for inguinal hernia in Byzantine times (A.D. 324-1453): first scientific descriptions. World J Surg.

[14] Mylonas AI, Poulakou-Rebelakou EF, Androutsos GI, Seggas I, Skouteris CA, Papadopoulou EC (2014). Oral and cranio-maxillofacial surgery in Byzantium. J Craniomaxillofac Surg.

[15] Sorta-Bilajac I, Muzur A (2007). The nose between ethics and aesthetics: Sushruta's legacy. Otolaryngol Head Neck Surg.

[16] Sperati G (2009). Amputation of the nose throughout history. Acta Otorhinolaryngol Ital.

[17] Whitaker IS, Karoo RO, Spyrou G, Fenton OM (2007). The birth of plastic surgery: the story of nasal reconstruction from the Edwin Smith Papyrus to the twenty-first century. Plast Reconstr Surg.

[18] Brain DJ (1993). The early history of rhinoplasty. Facial Plast Surg.

[19] Brown PF (1996). Venice & Antiquity: The Venetian Sense of the Past. https://books.google.com/books?hl=en&lr=&id=zxR7E2VTVagC&oi=fnd&pg=PR11&dq=Venice+%26+Antiquity:+The+Venetian+Sense+of+the+Past&ots=wdqdW0VtXc&sig=Xa5YO_tmYljRx2zpNPA9KZ1LPNI#v=onepage&q=Venice%20%26%20Antiquity%3A%20The%20Venetian%20Sense%20of%20the%20Past&f=false.

